# Predictive accuracy of the Violence Risk Assessment Checklist for Youth in acute institutions: A prospective naturalistic multicenter study

**DOI:** 10.1192/j.eurpsy.2025.3

**Published:** 2025-01-13

**Authors:** Anniken Lucia Willumsen Laake, John Olav Roaldset, Tonje Lossius Husum, Stål Kapstø Bjørkly, Carina Chudiakow Gustavsen, Sara Teresia Grenabo, Øyvind Lockertsen

**Affiliations:** 1 Faculty of Health Sciences, Department of Nursing and Health Promotion, Oslo Metropolitan University, Oslo, Norway; 2 Centre for Research and Education in Forensic Psychiatry, South Eastern Norway Regional Health Authority, Oslo University Hospital, Oslo, Norway; 3 Faculty of Health Sciences and Social Care, Molde University College, Molde, Norway; 4 Department of Child and Adolescent Psychiatry, Oslo University Hospital, Oslo, Norway; 5 Youth Acute Child Welfare Institution, Oslo Municipality, Oslo, Norway

**Keywords:** emergency psychiatry, residential youth care, violence risk screening, V-RISK-Y, youth violence

## Abstract

**Background:**

Acute health and social services for children and adolescents often struggle with youth aggression and violence. Early identification of violence risk during institutional stay can help prevent violent incidents. As such, this study assessed the predictive accuracy of the Violence Risk Assessment Checklist for Youth (V-RISK-Y) aged 12–18 in two different juvenile settings providing 24-hour services for youth. Institutions were included from child and adolescent inpatient psychiatry and residential youth care under child protective services.

**Methods:**

A prospective, naturalistic observational study design was employed. V-RISK-Y was administered for youth admitted to four acute inpatient psychiatric units and four acute residential youth care institutions. Incidents of violence and threats during the youth’s stay were registered by institutional staff. In total, 517 youth were included in analyses, 59 of whom were registered with at least one incident of violence or threats during their stay. Area under curve (AUC) and logistic regression analyses were used to assess predictive accuracy and validity of V-RISK-Y.

**Results:**

For the overall sample, V-RISK-Y had good predictive accuracy, and the sum score of V-RISK-Y significantly predicted registered violent incidents. Stratified analyses indicated good predictive accuracy of V-RISK-Y for the inpatient units, but not for the residential youth care institutions.

**Conclusions:**

Findings imply that V-RISK-Y is accurate in identifying violence risk for youth admitted to inpatient psychiatric units but has limited predictive accuracy in residential youth care institutions. Future research should explore approaches to correctly identify violence risk in residential care settings.

## Introduction

Aggressive and violent behavior among youth pose a major concern for youth inpatient psychiatric units [1, 2] and residential youth care institutions [3-6]. Violence in institutional settings adversely impacts both youth and staff [2], and it is important to identify individuals at high risk for violence early during the institutional stay to prevent violent incidents [7, 8]. Reported prevalence of violent incidents in institutional settings varies. A meta-analysis on adult inpatient psychiatric patients from high-income countries found that almost one in five patients in acute psychiatric units had violent incidents during institutional stay [9]. For youth, there are some discrepancies in prevalence findings. One study found that 23.1% of youth admitted to inpatient psychiatric units had at least one incident of physical aggression during their stay [10], another 28.4% [7], whereas a third found a rate of violence among children and adolescent patients in psychiatric care of 32.5% [2]. Further, in residential care settings for youth, client-perpetrated violence is a major challenge [3, 11], with up to 81% of staff reporting experiences of violence [5, 12]. Violence risk assessments are used to inform choices about treatment and identify appropriate interventions [1, 13] and can help de-escalate aggressive behavior and mitigate violent events [14]. Further, routinely assessing violence risk is found to reduce use of coercion in adult psychiatric inpatient units, leading to enhanced safety for staff and patients [8, 15, 16].

The predictive accuracy of a risk assessment tool depends on the population and context in which the assessment is conducted [17, 18]. Although some violence risk assessment instruments are validated for use in other clinical settings, most instruments are developed within forensic contexts [19, 20]. For instance, a comparative systematic review on violence risk assessments found that the Structured Assessment of Violence Risk in Youth (SAVRY), designed for youth populations, had better predictive accuracy compared to instruments without a specific target population [17]. In addition to SAVRY [21], several other risk assessment instruments are developed specifically for youth and utilized in forensic and nonforensic settings [22, 23], including the Youth Level of Service/Case Management Inventory (YLS/CMI) [24] and the Short-Term Assessment of Risk and Treatability: Adolescent Version (START:AV) [25]. However, the utilization of these instruments is typically resource demanding, as they are time-consuming and often require specific training to use [13]. For instance, in a qualitative study assessing staff experiences implementing START:AV in a residential care facility for youth, the time needed to appropriately implement the structured risk assessment was reported as a barrier leading to staff resistance [26].

Characteristics of acute institutions for youth may preclude administration of extensive risk assessments. For instance, institutional stays have a high turnover rate [27] and are typically brief, with many stays lasting only a day or two [28]. Accordingly, quick decision-making about risk and risk management is required [29, 30]. Youth can be admitted at any time, and at point of admission available resources may not allow for comprehensive violence risk assessment; for instance, staff trained in violence risk assessments may be unavailable [31]. Consequently, opportunity for administering current violence risk assessments for youth is limited in these settings. Providing a method for rapidly identifying violence risk in acute mental health settings, briefer violence risk screening instruments have been developed and validated for adults in acute psychiatric units [32]. For instance, V-RISK-10 is a screener validated for adult populations in acute psychiatric inpatient units [33]. For adult inpatient populations, V-RISK-10 has good predictive accuracy for adult inpatient populations with area under the curve (AUC) values between .79 and .82 [34-36] and has been internationally recommended for violence risk screening in inpatient psychiatric settings [32].

However, despite the apparent need, efforts to develop comparable screening instruments for youth populations are sparse [31]. To our knowledge, the only validated instrument for rapidly identifying violence risk for children and adolescents is The Brief Rating of Aggression by Children and Adolescents (BRACHA), which was developed to assess violence risk in children and adolescents admitted to inpatient psychiatric care from the emergency department [37]. The Violence Risk Assessment Checklist for Youth (V-RISK-Y) aged 12–18 is a violence risk screening instrument based on V-RISK-10. V-RISK-Y was developed for use in acute youth institutions and designed to be quick and easy to use, even for untrained staff, in situations where conducting comprehensive violence risk assessments are not feasible [31]. Following the structured professional judgment (SPJ) tradition [38], the risk appraisal is based on all relevant available information (i.e., both item scores and clinical judgment). In V-RISK-Y, five items are kept from V-RISK-10, whereas five items are adapted to reflect characteristics of adolescents. Additionally, two items are added: one item assessing severe trauma and one item assessing the youth and/or guardians’ own perception of violence risk. As described in the pilot study, these items were added because previous studies have identified them as violence risk factors [31].

V-RISK-Y was piloted in an emergency inpatient psychiatric unit for youth in Norway, where results indicated good predictive validity (AUC = .76) and acceptable internal consistency (Cronbach’s alpha = .78) [31]. A subsample of youth had been scored with both V-RISK-Y and V-RISK-10 at admission, and comparison showed that V-RISK-Y (AUC = .72) had slightly better predictive accuracy than V-RISK-10 (AUC = .70) [39]. Further, a case vignette study assessing the interrater reliability of V-RISK-Y found good interrater reliability for the V-RISK-Y sum score and low-moderate-high risk level [40]. However, there were discrepancies in agreement on item level, with particularly low interrater reliability for severe mental health symptoms and suspicion. Findings were interpreted to support the SPJ tradition the instrument is developed within, as the interrater reliability for the low-moderate-high risk level was excellent [40]. Raters included staff from both child and adolescent psychiatric units and child protective services, and comparisons of staff groups (i.e., physician/psychologist and professions that do not require graduate education) from the two settings yielded similar results [40].

### Study aims

The objective of the current study is to assess the predictive accuracy of V-RISK-Y for youth in two types of acute institutions providing 24-hour care for youth, namely acute inpatient psychiatric units and acute residential youth care units under child protective services. The study aims to assess predictive accuracy of V-RISK-Y for violent incidents, as well as for violence type and severity. Further, the study aims to compare predictive accuracy of V-RISK-Y between the two types of institutions to assess whether V-RISK-Y performs differently across settings.

## Methods

### Design, setting, and data collection

This naturalistic, prospective observational multicenter study was conducted in Norway at four emergency psychiatric inpatient units for youth and four residential youth care units with a total of 51 beds, spanning two of four health regions in Norway. The inpatient units provide inpatient services for children and adolescents with symptoms of mental health disorders of a severity that precludes outpatient treatment. Staff at the inpatient psychiatric units consist of physicians/psychiatrists, psychologists, and milieu therapists (e.g., nurses; social workers). Doctors and psychologists are only present during the daytime shift. The residential youth care institutions are custodial facilities within the child protective services system and provide housing and support for youth unable to live at home due to caregiver situation or behavioral issues. There is a high prevalence of mental health symptoms among youth in residential youth care, and frequent need for mental health care [41]. Staff at the residential care institutions consist largely of individuals with a social science background (i.e., milieu therapists and social workers only), and no physicians/psychologists. Thus, while the two types of institutions have different mandates, there is overlap in the need for mental healthcare services between the youth populations served by the inpatient units and residential youth care institutions. Both settings provide 24-hour emergency care for vulnerable children from the general population and are not forensic or correctional institutions. The psychiatric inpatient units are governed by the Mental Health Care Act. Residential care institutions under child protective services are governed by the Child Welfare Act, under which youth can be placed with legal basis in either a custodial or behavioral paragraph. Youth can be admitted to the institutions voluntarily or compulsory.

The planned data collection period was 1 year; however, the covid pandemic led to variations in start date and minor length of collection (range: 12–14 months). Overall, data were collected from September 2021 to April 2023. All youth aged 12–18 admitted to the participating institutions during the data collection period were included in the study.

The study received ethical approval from the Regional Committee for Medical and Health Research Ethics (REK ID: 218444). Exemption was granted from acquiring informed consent to participate from youth and/or their guardians. Youth and guardians received written information about the study and were informed about their right to withdraw from participation. The youth and/or their guardians had no further contact with the study.

### Sample

**Figure 1.**
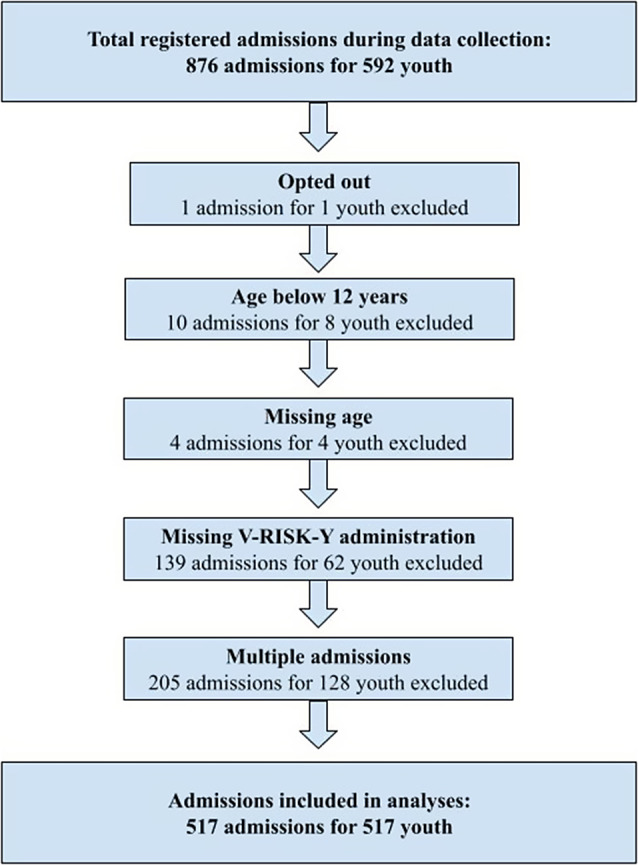
Flow chart for included cases.

Sample characteristics for the included youth (*n* = 517) are displayed in Table 1. Figure 1 displays applied exclusion criteria and exclusions. Only one admission per youth was included in analyses, so that any person would only count once in analyses. For included youth with registered violent incidents (*n* = 59, 11.4%) and multiple admissions, the first admission with registered violence was included in analyses. For youth without violent incidents and multiple admissions (*n* = 458), the first admission with complete V-RISK-Y scorings was included. Of the 59 youth registered with violent incidents (range 1-38), 35 (59%) were registered with more than one violent incident. Seven youth (12%) had five incidents or more, and one had 38 incidents. The mean age was 15.0 for girls and 15.6 for boys (*t* = −4.455, *p* < .001).

**Table 1.** Sample characteristics by type of institution and sex

	Inpatient psychiatric units (*n* = 355)		Residential youth care (*n* = 162)		***p*** ^a^	Total (*n* = 517)
	**Girls** ^b^ **(*n =* 267)**	**Boys** ^b^ **(*n =* 87)**	**Girls** ^b^ **(*n = 95*)**	**Boys** ^b^ **(*n = 66*)**	**<.001** ^c^	
Age, mean (median)	15.0 (15.0)	15.8 (16.0)	15.0 (15.0)	15.3 (16.0)	.520^d^	15.2 (15.0)
Reason for admission, *n* (%)^e^						
Suicidal	178 (68.7)	38 (45.8)	3 (3.4)	1 (1.6)	**<.001** ^d^	221 (44.6)
Eating disorder	31 (12.0)	0 (0.0)	0 (0.0)	0 (0.0)	**<.001** ^d^	31 (6.3)
Behavior	9 (3.5)	7 (8.4)	31 (35.6)	36 (56.3)	**<.001** ^d^	84 (17.0)
Assessment	26 (10.0)	21 (25.3)	1 (1.1)	0 (0.0)	**<.001** ^d^	48 (9.7)
Care/custody	0 (0.0)	0 (0.0)	52 (59.8)	27 (42.2)	**<.001** ^d^	79 (16.0)
Other	15 (5.8)	17 (20.5)	0 (0.0)	0 (0.0)	**<.001** ^d^	32 (6.5)
Days in institution, mean (median)	11.7 (4.0)	12.3 (5.0)	31.7 (15.0)	29.6 (20.5)	**<.001** ^c^	17.8 (5.0)

a Analyses comparing inpatient psychiatric units and residential youth care institutions.

b Missing data: 1–2%.

c Independent samples *t*-test.

d Chi square.

e Missing data: 4.3%.

### Procedure

Prior to project implementation, staff participated in a 3-hour introduction seminar where they received information about V-RISK-Y and the research project. No specific training in V-RISK-Y administration was provided, as the instrument aims to be self-explanatory and employable without training. Upon youths’ institutional admission, V-RISK-Y was administered by staff. For this study, V-RISK-Y was completed for research purposes and was not at the time fully integrated as routine assessment in the participating institutions. The recommendation was to do the scoring interdisciplinary if feasible. The form was filled out manually on paper. The youth and/or caregivers were not present during scorings. Instructions were to score V-RISK-Y based on available information (e.g., intake session, medical records, referral), without efforts to obtain additional information. Typically, V-RISK-Y was scored following the intake session, where staff had an opportunity to collect relevant data (e.g., assess youth/guardian’s perception of violence risk). Further description of scoring instructions is included in the pilot study [31] and on the V-RISK-Y form (accessible at https://sifer.no/verktoy/v-risk-y/). During the youth’s stay, after V-RISK-Y was administered, violent incidents were recorded by staff in the Violence/Threats registration form (VT Form). Registrations of violence and threats in the VT Form were recorded by staff present at the time of the incident.

### Measures

#### V-RISK-Y for youth aged 12–18

V-RISK-Y consists of 12 items scored as “no,” “don’t know,” “maybe/moderate,” or “yes.” When items are scored, the categorical risk level is indicated as “low,” “moderate,” or “high.” V-RISK-Y was used as baseline measure to identify violence risk during the youths’ institutional stay. V-RISK-Y can be downloaded for free from www.sifer.no.

#### VT Form

In line with previous studies [e.g., 42, 43], violence was defined as attacks against another person with the intent of causing harm and registered as severe (resulting in physical injury) or nonsevere (not resulting in physical injury). Threats were included in the definition of violence. The VT Form was used to register events of threats and/or violence during the participating youths’ stay in the institution. This form has also been used in comparable studies [e.g., 31]. Threats were categorized in the VT Form as verbal (threats directed toward a person in physical proximity, or severe threats on social media/texts) or physical (threatening movements/gestures directed towards a person in physical proximity). Definitions of each category of violence and threats were included in the VT Form. Registered incidents of threats and violence were used as outcome measure.

### Statistical analysis

SPSS 29 was used for all statistical analyses, except a Poisson regression model that was conducted in R. Statistical significance level was set to 0.05. Analyses were conducted and reported in line with the Risk Assessment Guidelines for the Evaluation of Efficacy (RAGEE) statement, which provides guidelines for studies on predictive accuracy of risk assessments [44]. Power analyses were conducted prior to the study, based on data from the pilot study [31]. Standard power analyses were calculated with 5% significance level and 80% power. For power analyses, .65 was considered the lowest clinically significant AUC value.

#### Baseline variables

To calculate the sum score for statistical analyses, the V-RISK-Y item scores were coded as “no” = 0, “don’t know” = 1, “maybe/moderate” = 2, and “yes” = 3. This coding is in line with previous research on V-RISK-Y and V-RISK-10 [e.g., 31, 40, 45]. “Don’t know” scores were weighted in analyses, based on findings from research on V-RISK-10 that unknown presence of item is associated with heightened risk [45]. The sum score of V-RISK-Y was calculated by adding the scores of the 12 items (0–36). The overall risk assessment was coded as “low” = 1, “moderate” = 2, and “high” = 3. Missing values for item scores on V-RISK-Y were replaced by mode imputation (25 missing values were replaced). Because the proportion of missing values were only 0.4%, and no items had missing values above 1.4%, the imputation did not impact results. This method ensures that all publications from this dataset include the same values, also those studies more vulnerable to missing data. Cronbach’s alpha was used to assess the internal consistency of V-RISK-Y.

#### Outcome variables

In main analyses of predictive validity and estimations of effect sizes, all recorded events of threats and violence were combined into a dichotomous variable (violence/no violence). To assess predictive validity for different categories, registered incidents of violence and threats were categorized as “threats” (physical and verbal threats); “nonsevere violence” (nonsevere threats and violence; nonsevere violence); and “severe violence” (severe threats and violence; severe violence). These categorizations were made to maintain statistical power. Violence categories were coded as dichotomous variables with type of violence/no violence.

#### Predictive accuracy, validity, sensitivity, and specificity

Predictive accuracy was determined by area under the curve of the receiver operating characteristics (AUC-ROC). AUC values of 0.7–0.79 are commonly interpreted as acceptable; 0.80–0.89 as good; and 0.9–1.0 as excellent [46]. In violence risk assessment research, a “glass ceiling” effect has been identified for AUC values [47]. Thus, violence risk assessments will at best have “acceptable” discriminatory abilities according to common benchmarks. The relationship between AUC and Cohen’s *d* can give a fuller picture of the strength of the AUC values [48]. Cohen’s *d* gives information about the size of the difference in V-RISK-Y score between youth with and without registered violent incidents. An AUC value of .714 converts to Cohen’s *d* of 0.8, which is typically considered a large effect size [48]. Accordingly, AUC values of .714 and above will be interpreted as indicating good predictive accuracy. An AUC value of approximately .802 converts to Cohen’s *d* of 1.2, which is considered very large effect size. AUC values were calculated for the sum score of V-RISK-Y, as well as for the sum score of the first 10 items (VY10), the first 11 items (VY11), and the first 10 items and item 12 (VY10 + 12). These analyses were conducted to assess the predictive accuracy of the instrument with none, one or both new items (*Trauma* and *Own Perception*) included in analyses, to see whether these items (that are not included in V-RISK-10), adds to the accuracy of the instrument.

The cutoff value for the V-RISK-Y sum score was determined from coordinate points on the ROC curve. Sensitivity, specificity, positive predictive value (PPV), and negative predictive value (NPV) were calculated based on the cutoff of V-RISK-Y sum score producing the highest sum of sensitivity and specificity where sensitivity above 80% was maintained. Sensitivity is the proportion of youth with registered violent incidents who were correctly identified by V-RISK-Y, and specificity is the proportion of youth not registered with violent incidents who were correctly identified by V-RISK-Y. PPV is the probability that youth with registered violent incidents are correctly identified by V-RISK-Y, and NPV is the probability of correctly identifying youth without registered violent incidents. A value of 1 indicates perfect sensitivity, specificity, PPV, and NPV.

Univariate and multiple logistic regression was used to calculate predictive validity of V-RISK-Y sum score, using Exp (B) as odds ratio (OR) for registered violent incidents. Analyses were conducted for all registered violent incidents grouped together, as well as for categories of violence (threats, nonsevere violence, and severe violence). Logistic regression analyses were also conducted for VY10, VY11, and VY10 + 12. Chi square was used to indicate total variance for each step in the multiple regression model, and explained variance was indicated by Cox & Snell *R^2^* (lower limit) and Nagelkerke *R^2^* (upper limit).

## Results

### Registered violent incidents

Of the included youth, 59 (11.4%) were recorded with at least one violent incident. There were 163 registered incidents of violence and threats among the included youth, ranging from 1 to 10 events per youth. There were significantly more youth with recorded violent incidents in the residential youth care institutions (*n* = 30; 18.5%) as compared to the inpatient units (*n* = 29; 8.2%; *χ^2^* = 11.7 (1), *p =* <.001). Overall, significantly more boys (*n* = 32, 20.9%) than girls (*n* = 27, 7.5%) were registered with violent incidents (*χ^2^* = 19.1 (1), *p =* <.001). Table 2 displays the registered violent incidents included in analyses by sex within type of institution, and by assigned low-moderate-high risk level.

**Table 2.** Registered episodes of violence and threats by type of institution

	Inpatient psychiatric units (*n* = 355)		Residential youth care (*n* = 162)		***p*** ^a^	**Total, *n* (%)** ^b^ **(*N* = 517)**
	Girls (*n =* 267)	Boys (*n =* 87)	Girls (*n =* 95)	Boys (*n =* 66)		
**Registered violent episodes, *n* (%)**	18 (30.5)	11 (18.6)	9 (15.3)	21 (35.6)	**<.001**	59 (100.0)
Threats (verbal and physical)	4 (6.8)	3 (5.1)	3 (5.1)	7 (11.9)	**.008**	17 (28.9)
Nonsevere violence or nonsevere violence and threats	13 (22.0)	5 (8.5)	4 (6.8)	8 (13.6)	.193	30 (50.8)
Severe violence or severe violence and threats	1 (1.7)	3 (5.1)	2 (3.4)	6 (10.2)	**.005**	12 (20.3)
**Low-moderate-high risk**						
Low risk	4 (6.8)	1 (1.7)	4 (6.8)	5 (8.5)	.249	14 (23.8)
Moderate risk	11 (18.6)	5 (8.5)	3 (5.1)	11 (18.6)	.514	30 (50.8)
High risk	3 (5.1)	5 (8.5)	2 (3.4)	5 (8.5)	.708	15 (25.4)

a Chi square tests comparing inpatient psychiatric units and residential youth care institutions.

b Missing data for 0–1.3% of admissions.

V-RISK-Y sum scores (0–36) had a mean of 13.95 and a median of 13.00. The sum score was significantly lower for girls (*M* = 13.10; median = 13.00) than for boys (*M* = 16.01; median = 15; *t* = −4.33; CI: −4,23, −1.59; *p* < .001). For the two types of institutions, the sum score was significantly lower for inpatient units (*M* = 13.08; median = 12.00) as compared to residential youth care institutions (*M* = 15.85; median = 15.00; *t* = 4.19; CI: 1.47, 4.07; *p* < .001).

### Predictive accuracy

#### AUC values

AUC values for the full sample, as well as stratified by sex and type of institution, are displayed in Table 3. AUC values for the abbreviated sum scores (i.e., VY10, VY11, and VY10 + 12) are displayed in Table 4.

**Table 3.** Area under the curve (AUC) values for V-RISK-Y and the low-moderate-high risk category

	Sum score		Low-moderate-high risk category	
	AUC (95% CI)	*p*	AUC (95% CI)	*p*
Full sample	.762 (.700, .824)	**<.001**	.770 (.702, .837)	**<.001**
Girls	.735 (.635, .835)	**<.001**	.757 (.653, .860)	**<.001**
Boys	.747 (.661, .833)	**<.001**	.737 (.641, .834)	**<.001**
Inpatient psychiatric units (IPU)	.818 (.747, .889)	**<.001**	.823 (.739, .907)	**<.001**
Youth residential care (RYC)	.670 (.560, .779)	**.004**	.686 (.577, .795)	**.002**
Girls IPU	.792 (.697, .888)	**<.001**	.800 (.685, .914)	**<.001**
Boys IPU	.843 (.738, .948)	**<.001**	.844 (.726, .961)	**<.001**
Girls RYC	.598 (.367, .828)	.337	.665 (.461, .870)	.104
Boys RYC	.624 (.480, .768)	.107	.615 (.466. .764)	.137

**Table 4.** area under the curve values for abbreviated V-RISK-Y sum scores

	AUC (95% CI)	*p*
VY10 sum score (10 first items)	.751 (.688, .814)	**<.001**
VY11 sum score (11 first items)	.745 (.681, .809)	**<.001**
VY10 + 12 sum score (10 first items and item 12)	.767 (.707, .828)	**<.001**

#### Sensitivity and specificity

Maintaining sensitivity of >80%, the best cutoff for V-RISK-Y for the full sample was 12.5. With this cutoff, V-RISK-Y had sensitivity of .83, specificity of .52, PPV of .17, and NPV of .96.

#### Logistic regression analyses

Results from logistic regression analyses on V-RISK-Y and violent events, stratified by type of institution, are displayed in Table 5. In univariate logistic regression analyses with registered violent events as outcome, sex was significant (OR = 3.281; CI: 1.89, 5.70; *p* < .001) and included in multivariate analyses. Age was not significant (OR = .995; CI: .83, 1.20; *p* = .959) and thus not included. Poisson regression model for V-RISK-Y sum score and number of violent events was not significant (β = −.008; *p =* .498). Length of stay was significantly associated with violent incidents, with an increase in odds for violence by 1.02 (95% CI: 1.01, 1.03; *p* < .001) per additional day in the institution. Stratified analyses were significant only for inpatient units (OR = 1.03; 95% CI: 1.02, 1.04; *p* < .001) and insignificant for residential youth care institutions (OR = 1.01; 95% CI: 1.00–1.02, *p* = .058). When length of stay was added to the model, the OR remained the same for V-RISK-Y sum score, but the explained variance increased for inpatient units (13.5–31.5%) and for residential care institutions (12.0–19.5%). Table 6 displays findings for logistic regression analyses on type of violence.

**Table 5.** Stepwise multivariate logistic regression analyses stratified by type of institution

	Inpatient psychiatric units					Residential youth care				
	OR (95% CI)	*p*	Total variance^a^	*p*	Explained variance^b^	OR (95% CI)	*p*	Total variance^a^	*p*	Explained variance^b^
**Step 1**										
Sex (ref = girls)	2.00 (.906, 4.42)	.086	2.8 (1)	.095	0.8–1.8%	4.46 (1.89, 10.54)	**<.001**	12.7 (1)	**<.001**	7.6–12.3%
**Step 2**										
Sex (ref = girls)	1.51 (.636, 3.57)	.352				3.63 (1.50, 8.80)	**.004**			
V-RISK-Y sum score	1.16 (1.09, 1.23)	**<.001**	32.2 (2)	**<.001**	8.7–20.1%	1.07 (1.00, 1.14)	**.046**	16.8 (2)	**<.001**	9.9–16.0%

a For each step, Chi square (degrees of freedom).

b For each step, Cox & Snell *R^2^* - Nagelkerke *R^2^.*

**Table 6.** Logistic regression analyses for V-RISK-Y sum score and violence categories controlled for sex

	OR (95% CI)	*p*	Total variance^a^	*p*	Explained variance^b^
	**Threats (verbal and physical; *n* = 17)**				
**Step 1**					
Sex (ref = girls)	3.55 (1.32, 9.50)	**.012**	6.4	**.011**	1.2–4.9%
**Step 2**					
Sex (ref = girls)	2.56 (.927, 7.09)	.070			
V-RISK-Y sum score	1.12 (1.04, 1.21)	.002	16.8	**<.001**	3.2–12.8%
	**Nonsevere violence and threats (*n* = 30)**				
**Step 1**					
Sex (ref = girls)	1.88 (.892, 3.98)	.097	2.7	.103	0.5–1.4%
**Step 2**					
Sex (ref = girls)	1.48 (.681, 3.20)	.325			
V-RISK-Y sum score	1.08 (1.03, 1.14)	**.002**	12.4	**.002**	2.4–6.6%
	**Severe violence and threats (*n* = 12)**				
**Step 1**					
Sex (ref = girls)	7.48 (2.00, 28.0)	**.003**	10.8	**.001**	2.1–10.4%
**Step 2**					
Sex (ref = girls)	5.08 (1.31, 19.7)	**.019**			
V-RISK-Y sum score	1.15 (1.05, 1.25)	**.002**	21.9	**<.001**	4.2–21.0%

a For each step, Chi square (degrees of freedom).

^b^ *For each step, Cox & Snell R2 - Nagelkerke R^2^.*

### Internal consistency

The internal consistency for V-RISK-Y with all 12 items included was .791 (95% CI: .76, .82; *p* < .001). If one item was deleted, the internal consistency was highest for item 3 (substance use; Cronbach’s alpha = .795), and lowest for item 2 (threats; Cronbach’s alpha = .753).

## Discussion

### Main findings

For the overall sample, the predictive accuracy of V-RISK-Y for violent events was acceptable for the sum score and for the low-moderate-high risk level (see Table 3). Predictive accuracy was better in inpatient units compared to the residential youth care, and the highest predictive accuracy of V-RISK-Y was found for boys in inpatient units (see Table 3). When controlled for sex, V-RISK-Y score accounted for 9–20% and 10–16% of variance in registered violent incidents in inpatient units and residential youth care, respectively (see Table 5). When stratified by sex, AUC values were not significant for the residential youth care institutions. V-RISK-Y score was significantly associated with higher odds for all types of assessed violence (i.e., threats; nonsevere violence; and severe violence; see Table 6).

### Overall predictive accuracy

AUC values indicate good predictive accuracy for the sum score and the low-moderate-high risk level for the overall sample and for the youth in the inpatient units. When length of stay was controlled for, the explained variance of V-RISK-Y increased for both institutions. Length of stay is commonly associated with increased violence [e.g., 49]. Accordingly, we found higher rates of violence in the residential care institutions compared to the inpatient units. At the same time, we know that violence risk is increased early during institutional stay and that violence may lead to longer stays [e.g., 7, 9]. Thus, these findings should be interpreted with caution. When sensitivity is prioritized, as is common in violence risk assessment research, it typically leads to lower specificity [e.g., 50]. Further, in this study, the PPV value (.17) is low, indicating that a low percentage of youth with a score above the cutoff will be registered with violent incidents during their institutional stay. This implication may, in part, be due to the quite low prevalence of violence (11.4%) found in this study, which is associated with low PPV [51].

Analyses for the V-RISK-Y sum score with and without inclusion of the items *Trauma* and *Own Perception* produced different results (see Table 4). Including the *Trauma* item in analyses led to a relative decrease in predictive accuracy. Thus, while severe childhood trauma is an identified risk factor for violent behavior in youth [52], it did not increase predictive accuracy in this study. Similar findings were produced in the V-RISK-Y pilot study, where it was hypothesized that the high proportion of youth in the inpatient units with experiences of severe trauma reduced the discriminatory abilities of this item [31]. As suggested by Roaldset and colleagues [31], the *Trauma* item might be more relevant for predicting violence in a community or outpatient population where there is a lower frequency of severe trauma experiences. It is also possible that trauma characteristics are relevant; a study exploring types of adverse events in childhood and risk profiles supports a more granular as opposed to cumulative approach in linking trauma characteristics to risk profiles [53].

Conversely, the inclusion of *Own Perception* resulted in increased predictive accuracy. These findings are in line with contemporary approaches promoting user involvement and participation [e.g., 54]. Users own assessment of risk is not commonly included in risk assessments, despite an established positive association between own endorsement of risk and violent behavior [e.g., 55, 56].

### Differences between types of institutions

While it was hypothesized that similarities between the youth populations served by inpatient units and residential youth care would yield similar findings of predictive accuracy of V-RISK-Y for the two types of institutions, findings indicate substantial differences between the two institution types. Different characteristics of institutions and the youth populations they serve could partially help explain the discrepancies in predictive accuracy (see Table 3). For instance, the mandates of the two types of institutions differ (i.e., treatment versus custodial). These mandates are reflected in reason for admission (see Table 1), with suicidality being the most common reason for admission to inpatient units and custodial or behavioral issues being the most common reasons for placement in residential youth care. Youth in residential care had higher scores on V-RISK-Y and more registered violent incidents as compared to the inpatient units. It is possible that the youth in the residential care institutions have higher rates of conduct disorder with a tendency towards violent behavior as compared to youth in the psychiatric inpatient units.

Placement in residential care is considered a last resort, following failed attempts to intervene at home [57]. Children in residential care in Nordic countries have more disadvantaged backgrounds compared to their peers [58]. Children and adolescents under residential care are commonly from marginalized homes [59] and often have experiences of maltreatment and psychosocial struggles [60]. Violence exposure is a risk factor for aggressive and violent behavior [61, 62], which might partially explain the higher rates of violence in the residential youth care institutions. It is possible that the complexity of psychosocial challenges is greater for this youth population as compared to youth in the psychiatric inpatient units. Perhaps, this complexity is not captured by the current version of V-RISK-Y, decreasing the ability of the instrument to discriminate between youth with and without violent behavior. Further, because so many risk factors are typically present, they may not in themselves be associated with higher violence risk by staff. As displayed in Table 2, the residential youth care institutions had the highest proportion of youth who were registered with violent incidents assessed with “moderate” risk, and the highest rates of violent incidents. As per the IRR study, there were not substantial differences between the staff groups in the overall V-RISK-Y assessment. However, it is possible that some of the items are more relevant in psychiatric units (such as severe mental symptoms) and therefore does not work as well in the residential care institutions.

These hypotheses do not, however, preclude staff differences in knowledge and expertise in violence prevention and/or de-escalation strategies. Given their different contexts and functions, staff composition between the two types of institution differ. Residential youth care institutions are more heavily staffed by social workers as opposed to healthcare workers and do not have physicians or psychologists on staff. This staff composition may be associated with lower competency in violence risk screening and/or strategies applied in managing violence risk. However, one study assessing the impact of contextual factors on aggressive and violent behavior in residential care for youth found that contextual factors had little impact on aggression [6]. This finding could illustrate a high degree of behavioral challenges that are not mediated by contextual aspects of the institutional stay and that differ between the youth admitted to inpatient psychiatric care and those placed in residential care.

V-RISK-Y is based on V-RISK-10, which was developed for psychiatric inpatient populations. These origins may contribute to V-RISK-Y being more adapted to the youth in inpatient psychiatric units. Aggression and violent behavior are not necessarily symptoms of a mental health disorder. A youth with behavioral challenges related to aggression that caregivers are unable to manage at home, and that are not directly a symptom of a mental health disorder, would not be hospitalized in a psychiatric unit. Instead, child protective services would more likely be contacted and the youth would be moved to their custody if necessary. Thus, the residential care units see more of the youth with behavioral difficulties as the main challenge, rather than mental health. Versions of V-RISK-10 have been adapted to specific settings, such as V-RISK-POL, for use at police stations [63]. Generally, few structured risk assessment instruments are developed specifically for acute child welfare services, and tools adapted to this population have been requested [64]. Perhaps, risk factors specific to the residential care institutions should be mapped out to inform a screening instrument better tailored to this setting.

### Sex differences

Results in predictive accuracy were similar between girls and boys for the overall sample. In analyses stratified by type of institution and sex, sex discrepancies were found in predictive accuracy (see Table 3). V-RISK-Y had better predictive validity for boys than for girls within both institution types. For inpatient units, predictive accuracy was better for boys than girls but good for both sexes. Previous studies have found sex differences in risk profiles [65], and one possible explanation for these results is that the instrument better captures risk factors characteristic for boys. However, the reason for admission between sexes also varied greatly, with many girls being admitted to inpatient units for internalizing problems such as self-harm/suicidality (see Table 1). For residential youth care institutions, however, AUC values were insignificant for both sexes. Further, in logistic regression analyses stratified by type of institution, sex was a highly significant variable in residential youth care institutions, where it accounted for 8–12% of the explained variance in registered violence (see Table 5). As displayed in Table 2, only 16 (17%) of the girls were registered with violent incidents during their stay in residential care units, compared to 36 (55%) boys. It is possible that the girl group was too small to yield significant results. Because the majority of the violent incidents in the residential care institutions are registered for boys, it is possible that they reflect the overall findings for the residential care units. A higher proportion of boys than girls were placed in residential care for behavioral challenges, and boys’ violent behavior was of a higher severity than for girls. When analyzed as a whole, AUC values are significant, but low. While most participants were girls, the residential youth care had a higher proportion of boys than the inpatient units. It is possible that this ratio, combined with higher mean V-RISK-Y score, contributed to decreasing the instrument’s discriminatory ability. Most severe violent incidents were registered for boys (70%). Of the violence categories, best predictive accuracy of V-RISK-Y was found for severe violence, which could explain why the instrument seems more accurate in predicting violence for boys in this study. Further, cultural gender expectations may contribute to appraisals of violence risk. For instance, male sex is stereotypically more associated with overt aggression [e.g., 66, 67].

### Limitations

Given the naturalistic nature of the study, it was not possible to control for external variables. The study took place in Norway, and generalizability of findings cannot be assumed to other countries or settings without further validation. Because of the limited number of demographic variables collected in this study (i.e., sex and age), more nuanced demographic information is not presented nor included in analyses. Baseline violence rates in participating institutions were unknown, and it is not clear whether the registered violent incidents are comparable to baseline for the participating institutions. This paper does not include descriptions or analyses of who incidents of violence or threats were directed toward (e.g., staff; peers). As such, the study does not provide insights about whether predictive accuracy of V-RISK-Y depends on the direction of violence and/or threats. Further, the administration of V-RISK-Y was not blinded, and the same staff groups responsible for scoring V-RISK-Y were also responsible for registering violent incidents if present when they occurred. Administering a violence risk screening as part of the study could potentially lead to implementation of preventive measures, preventing violent episodes from occurring. For instance, data from the V-RISK-Y pilot study showed that patients assesses with high risk were subjected to more restrictive means than those assessed with moderate risk [31]. Further, the sex composition of the sample, consisting of mostly girls, may have impacted the generalizability.

Finally, the data collection period partly overlapped with the covid pandemic, which could have impacted sample characteristics, findings, and generalizability. While covid research is still ongoing and the impact of the pandemic not fully established, factors as reason for admission may have been different during the pandemic. In Norway, there was a decline in physical and psychological health among adolescents [68]. During covid, there was an increase in internalizing mental health problems, whereas externalizing problems such as disruptive behavior remained unchanged [69]. Accordingly, there was a substantial increase in eating disorders during covid [70]. It is possible that these symptoms and disorders were more prominent in youth in the inpatient units during as compared to before covid.

### Implications and future research

This study contributes to establishing the overall predictive accuracy of V-RISK-Y. To our knowledge, V-RISK-Y is the first validated violence risk screening instrument for youth in acute 24-hour institutions. While overall predictive accuracy for violence is good, violence risk for youth in residential care, where violence is a major concern, must be captured better. Future efforts should explore the relevance of individual V-RISK-Y items in the residential youth care institutions and assess adaptations of the screening instrument in this setting.

V-RISK-Y should be validated in additional contexts to assess whether findings can be generalized to other settings and populations where violence risk screening is relevant, such as outpatient settings and inpatient settings outside of Norway. Future studies should look at how individual items of the instrument adds to predictive accuracy of violence risk, as well as assess potential sex differences in the accuracy of V-RISK-Y. Further, future research should establish whether the assumption that “don’t know” scores are associated with higher violence risk than “no” scores for V-RISK-Y is true, as has been found for V-RISK-10 [45].

## Data Availability

The data analyzed in this study are not publicly available and cannot openly be shared due to privacy laws and restrictions in ethical approval. Data are available from the authors upon reasonable request.
